# Supporting and Engaging Families: An Examination of Publicly-Funded Health Promotion Programs in the Intermountain West, USA

**DOI:** 10.3389/fpubh.2020.573003

**Published:** 2020-10-15

**Authors:** Lynneth Kirsten Novilla, Eliza Broadbent, Rozalyn Glade, AliceAnn Crandall

**Affiliations:** Department of Public Health, Brigham Young University, Provo, UT, United States

**Keywords:** health promotion, family engagement, family-focused programs, family impact analysis, family-centered programs

## Abstract

Public health programming efforts have traditionally focused on either an individualistic or population approach, neglecting the family as a setting for or partner in health promotion efforts. Due to the multi-faceted influence of families on individual health, family-focused, and family-friendly public health interventions are important to making lasting changes for individual and community health. The purpose of this study was to examine the degree to which health promotion programs in a state in the US Intermountain West involve and support families across four family impact principles: family engagement, family stability, family responsibility, and family diversity. A survey was completed by 67 health promotion administrators and practitioners from 12 out of 13 county health departments with additional responses from public health practitioners at the State Health Department. The results of the survey indicated that health promotion efforts were best at supporting family responsibility and a diverse group of families but were weaker in family engagement and family stability. Applying a more family-centered and family-focused approach to health promotion efforts can be achieved by employing interdisciplinary efforts and by taking advantage of tools like the Public Health Family Impact Checklist to intentionally engage and support families in programs and interventions.

## Introduction

Families have an important influence on individual health. Through families, individuals learn health habits and values (1), access healthcare based on the availability of family health insurance and decisions about receiving healthcare (2), and are genetically predisposed to health risks and benefits (3). Further, health is affected for better or worse by the social and emotional support system provided by family relationships, such as marriage relationships and the parent-child relationship (2, 4–6).

Public health frameworks, such as Healthy People 2020 and Public Health 3.0, call for an updated look into public health and how public health can better bridge communities and resources (7). Due to the influence of families on health, family-focused, and family-friendly public health interventions are one such way to bridge communities and are important to making lasting changes for individual and community health (8). Programs need to not only take into account individual family members, but also the family structure and contextual factors that inform family beliefs and activities. However, public health programs have traditionally had an individualistic perspective based on the medical model of health, and families are seldom incorporated into normal public health procedures like program planning and evaluation at the national or local level (9). Yet studies have found that promotion of health through policy and programs will be more beneficial and sustainable when family networks are engaged (10–12). For example, although children of single-parent families often have worse health outcomes, single parent families have the potential to be as successful in transmitting healthy practices to children as a household with two parents when the focus of an intervention is on involving each member of the family, allowing members of the family to take a more responsible and active role in participating in healthy behaviors (13–15).

Despite the importance of families to health, many public health professionals may be unsure how best to develop family-centered interventions. One way that health promotion programs can better have a family focus is to intentionally incorporate family impact principles into their programming. Family impact principles include family engagement, stability, responsibility, and diversity (9, 16). Family engagement refers to ensuring that there is a partnership between interventions and families while preserving family dignity and respecting family autonomy. Family stability focuses on encouraging stability within the family and recognizing the importance of family relationships to individual health and functioning. Programs strong in family responsibility deliver services that support and empower the functions that family should perform. Finally, family diversity is about understanding that families vary in their structure and characteristics and thus have unique needs that should be considered by the program. Programs that are strong in family diversity acknowledge and respect the differences of families and do not penalize families based on cultural or ethnic background, economic situation, family structure, geographic locale, presence of special needs, or religious affiliation. These guiding principles were originally developed by the Coalition of Family Organizations and revised by the Family Impact Institute in an effort to “shift the rhetoric from appreciating families to prioritizing them as worthy of study, investment, partnership, and political action” [(17), p. 263]. Two theories primarily form the theoretical framework for family impact principles. Ecological Family Systems Theory (17–19) purports that families are central to individual growth and development, and programs support or hinder the environment in which families function. Self-efficacy Theory (17, 20) states that families can better support healthy development in individual members when they have self-efficacy beliefs. Public health programs can help to build family self-efficacy by incorporating relational and participatory practices built on the foundations of respect and agency for families. Programs that support, recognize, and sustain family systems serve to promote the family's capacity to better health and development. However, when family impact principles are not considered, this hinders family autonomy and program success.

To help support the development and refinement of family-supportive and family-directed health promotion practice, a family impact checklist was developed specifically for public health practitioners (16). This checklist incorporates the family impact principles and uses them as a guide to determine the level of family impact of current health promotion programs. This tool allows health promotion specialists to have a more complete view of what family-centered approaches may be lacking in their current programming.

This study aims to examine how well-publicly-funded health promotion interventions in a state in the Intermountain West supported and involved families in their programming. Although anecdotal evidence suggests that health promotion programs do not adequately involve families, this has not been previously studied. Thus, this study will provide objective evidence on the strengths and weaknesses of health promotion programs in having a family-focus using the Public Health Family Impact Checklist (16). An evaluation of the present state of these programs in involving families can aid in improving their effectiveness by encouraging a family-centered and family-focused approach to program planning and evaluation.

## Materials and Methods

Following the receipt of institutional review board (IRB) approval, an online Qualtrics survey was sent via e-mail to all known health promotion workers at the 13 county health departments and state health department (*N* = 142) in a state located in the U.S. Intermountain West. These potential participants were selected based on their job title (i.e., health promotion directors, health educators) and whether they were involved in a health promotion intervention at the state or county level. Responses were received from 67 public health professionals who represented 12 out of 13 local county health departments and the state health department (47% response rate). Respondents came from both urban and rural regions of the state.

The majority of participants were involved in multiple projects relating to chronic disease control and prevention, injury prevention, substance abuse programs, and community health programs. Participants were asked to respond to the survey based on the program that they spent the most time on (or to select one if they spent equal time on multiple programs). Following receipt of the survey, participants were given a period of 1 week to complete the survey. Participants received a $10 Amazon gift card upon completion of the survey.

We used the Public Health Family Impact Checklist (16) to examine how well-health promotion programs support and involve families. The Public Health Family Impact Checklist contains 14 items examining how well-public health programs impact and support families. Response options were on a 3-point scale (0 “Not at all,” 1 “somewhat well, and 2 “Very well”) with higher scores indicating programs with better family support. Factor analysis confirmed the presence of three factors covering the principles of family engagement (α = 0.75), family responsibility (α = 0.79), and family stability (α = 0.73). Sample items included “To what degree are/were families involved in the development and planning of the program?” (family engagement); “How well does the program train and encourage staff and partners to support families to make their own decisions and respect family choices?” (family responsibility); and “How well does the program help families prevent health problems before they become serious and affect the family's ability to address the health issues/behavior?” (family stability). Additionally, two items examined aspects of family diversity (“How well does the program provide services that are available and accessible to diverse families and family types?” and “How well does the program address root causes of the health issue?”). However, because factor analysis did not confirm these items as a single factor the results are reported based on the individual items that represent different aspects of family diversity.

### Data Analysis

Data analyses were conducted in Stata 15. Item means were calculated for all 14 items. Additionally, average scores were calculated across the items for family engagement, family responsibility, and family stability.

## Results

The average score across the 14 items was 1.12 (*SD* = 0.38), indicating moderate support and involvement of families in health promotion programs. Table 1 contains the mean scores across items and family impact principles. Participants reported high variation in their levels of family engagement with scores ranging across items from 0.56 to 1.35 with an average score of 0.96 indicating somewhat low family engagement. Responses for family stability ranged from 0.84 to 1.4 with an average of 1.12 indicating moderate program support of family stability. Items relating to family responsibility ranged from 1.27 to1.36 with an average score of 1.31 indicating that programs had moderate (“somewhat well”) to high (“very well”) support of family responsibility. Additionally, programs reported a mean score of 1.45 on the family diversity item relating to program services' availability to a variety of families; the family diversity item relating to root causes had a mean score of 1.26.

**Table 1:** Summary of family impact principle results.

**Question**	**Mean (SD)**	**Very Well (2)**	**Somewhat Well (1)**	**Not at All (0)**
**Family impact principle: family engagement**				
To what degree are/were families involved in the development and planning of the program?	0.66 (0.64)	8%	47%	44%
How well does the program consider and provide services that support the whole family as a unit, including extended family where appropriate?	1.09 (0.67)	25%	58%	17%
How well does the program connect families to community resources related to the target health issue and help them become informed consumers of these resources?	1.35 (0.54)	42%	56%	3%
How well does the program help families build essential social support to address the health issue?	0.97 (0.61)	22%	69%	8%
How well does the program include families in program evaluation?	0.56 (0.68)	14%	31%	56%
Mean Family Engagement Score	0.96 (0.47)	–	–	–
**Family impact principle: family stability**				
How well does the program help families prevent health problems before they become serious and affect the family's ability to address the health issue/behavior?	1.41 (0.64)	56%	39%	6%
How well does the program help families maintain healthy routines when experiencing stressful conditions or times of change that may be related to the health issue/behavior?	1 (0.65)	22%	61%	17%
How well does the program recognize that major changes in family relationships and functionality can impact health, may extend over time, and require major support and attention?	0.84 (0.71)	14%	47%	39%
How well does the program facilitate healthy family relationships and recognize that individual development, well-being, and behavior change are profoundly affected by family relationships?	1.07 (0.66)	27%	56%	17%
Mean family stability score	1.12 (0.49)	–	–	–
**Family impact principle: family responsibility**				
How well does the program train and encourage staff and partners to support families to make their own decisions and respect family choices?	1.27 (0.65)	36%	53%	11%
How well does the program help families build the capacity to address their health needs without overstepping important boundaries necessary for healthy participant independence?	1.36 (0.64)	44%	47%	8%
How well does the program address participant needs and allow for a balance between work, family, and community commitments?	1.29 (0.60)	25%	67%	8%
Mean family responsibility score	1.31 (0.54)	–	–	–
**Additional items**				
Family Diversity: How well does the program provide services that are available and accessible to diverse families and family types?	1.45 (0.59)	53%	42%	6%
Root Causes: How well does the program address root causes of the health issue?	1.26 (0.63)	33%	58%	8%

*Higher scores denote more family support and involvement in programs*.

## Discussion

The purpose of this study was to conduct a preliminary examination of the current state of family support and involvement in health promotion programming. The results were mixed and demonstrated that while health promotion practitioners were family-focused in some ways, there is still more room for improvement. Health promotion programs were strongest on family inclusion for items that dealt with classic public health situations (e.g., addressing the root causes, supporting a diversity of families, connecting families to community resources, and early detection and prevention), but weaker in areas that may require some familiarity with family science (e.g., understanding how the family environment and circumstances affect health).

While public health focuses on the application of knowledge, skills, and competencies needed to perform essential public health services (21), the results of this study have shown that there is still a weakness in public health programs in applying family theories and family science methods to public health practice. The inclusion of family science principles such as human development and family system theories in public health training could help health promotion practitioners better understand and use the family as a partner in change (22). Other weaknesses that exist in current health promotion programming may stem from a lack of knowledge, training, and understanding about family impact principles and their ability to guide program planning and evaluation (23, 24).

Through this initial look at the strengths and weaknesses of current health promotion programming in involving the family, perhaps the most concerning result was that participants reported that their programs incorporated little involvement of families in the program planning and evaluation stages. The classic approach of health promotion has been a top-down view in connecting communities and families (25). However, Public Health 3.0 encourages the development of programs that bridge gaps between health promotion practitioners, programs, and communities. Creating a family-focus in community health programs is vital to finding more sustainable solutions to community and population problems because of the family's role in affecting and influencing the health outcomes of individuals within the family and the family as a unit (26). As families are recognized as the core to healthy individual member development, stronger and more family-centered, and family-focused programs and initiatives can be created that result in more effective and sustainable health solutions.

Studies have demonstrated that when families are engaged and supported in health promotion interventions, individuals will develop sustainable lifestyle changes. For example, a family-centered program to enhance family resilience was evaluated on its effectiveness among military families. The program utilized the family as a setting for change by asking military personnel and their immediate family members to participate in program class sessions where they were provided with family-level education on family stress reactions, family communication, family routines, and identifying family strengths (27). Through the involvement of families in their intervention program, researchers discovered that families experienced greater positive outcomes in resilience and family functioning. The results of this study indicated that there is value in employing more family-centered prevention programming as its effects on individuals, families, and consequently the larger population have a greater impact than has previously been acknowledged (27).

In public health practice, successes have been seen in family-centered programming for traditional public health problems, such as poor nutrition, diabetes prevention, and adolescent risk-taking behaviors that contribute to HIV. Multiple studies have demonstrated that when family practices, values, and responsibilities are included in public health interventions, there is a much higher likelihood of positive change for both individuals and families and lasting success (28–30). Programs like Healthy Home Offerings via the Mealtime Environment Plus (HOME Plus) or Strong African American Families (SAAF) are examples of programs that work with families to promote healthy eating habits in childhood to prevent obesity in adulthood. Evaluations of these programs have shown significant and sustained healthy eating habits for individual family members while also creating a healthier food environment in family homes (28–31).

Another example of a family-centered program is Let's Talk, which aims to prevent the contraction and transmission of HIV. In a study on the effectiveness of this program, the researchers found that in order for effective changes to take place for an individual who may have multiple risk factors, including poor psychological health and sexually risky behavior, they needed to include a parallel program for parents or caregivers. This resulted in a significant increase in the mental health of both adolescents and their parents or caregiver and in adolescent compliance to behaviors that reduce the likelihood of contracting or transmitting HIV (32).

As demonstrated by the results of this study, many health promotion programs are positively involving families, but there is room to improve the involvement of families in program planning and the application of family science theories and methods to public health practice. Tools such as the Public Health Family Impact Checklist (16) can help start a conversation about a family focus in health promotion programs (6) and guide programs to more intentionally engage and support families. Starting this conversation is an important step into eventually furthering new approaches in training current and future health promotion specialists on how to more effectively utilize the family in health promotion planning and evaluation.

## Limitations and Strengths

This study involved a small convenience sample of practitioners working on a variety of health promotion programs at the county and state level of one state in the Intermountain West. The results cannot be generalized to other states throughout the U.S. nor to communities outside of the U.S. Further research is merited to examine trends around the U.S. and in other countries. Different family impact principles may be more important depending on the issue each program is addressing. Additionally, responses from these public health experts were self-reported. Those who chose to respond may have been those who already valued the inclusion of a family-focus in public health, or they may have had differing perspectives based on where they worked (e.g., urban vs. rural communities) and whether they were an administrator, program manager or coordinator, or practitioners (e.g., health educator). However, we did not collect information based on job title and most respondents were from health departments from rural and urban areas, thus making stratification based on job title or type of community impossible. An additional limitation is that we did not examine potential determinants of why some programs involved or considered families more than others. Our analysis was a descriptive analysis of the current state of how well health promotion programs consider families across multiple family impact principles. Finally, we surveyed only practitioners working on publicly-funded health promotion efforts—the results do not include respondents from non-governmental organizations. Although these limitations exist, this study is the first of its kind to examine the state of current public health programs as it relates to intentionality in involving the family. Thus, further research can build off of these preliminary findings using larger sample sizes that allow for more stratification of results and causal statistical analyses. Although we have noted that many programs have found greater success as they have involved families in their programming, it would also be important to explore the benefits of a family-focus across a greater breadth of health promotion programs.

## Data Availability Statement

The datasets presented in this article are not readily available because The Institutional Review Board has not approved distribution of the raw, anonymized data set. Upon request from the corresponding author, approval will be applied for as appropriate. Requests to access the datasets should be directed to ali_crandall@byu.edu.

## Ethics Statement

The studies involving human participants were reviewed and approved by Brigham Young University Institutional Review Board. Written informed consent for participation was not required for this study in accordance with the national legislation and the institutional requirements.

## Author Contributions

LN and AC conceptualized the study. LN, RG, and AC designed and administered the survey. AC oversaw data collection and analysis. EB helped review the literature and wrote sections of the introduction and discussion. All authors were involved in the writing process of the manuscript and reviewed and approved the manuscript.

## Conflict of Interest

The authors declare that the research was conducted in the absence of any commercial or financial relationships that could be construed as a potential conflict of interest.
